# Postoperative Pulmonary Edema Conundrum: A Case of Negative Pressure Pulmonary Edema

**DOI:** 10.1155/2018/1584134

**Published:** 2018-09-23

**Authors:** Pramod K. Guru, Anjali Agarwal, Mario Pimentel, Diane C. McLaughlin, Vikas Bansal

**Affiliations:** Department of Critical Care Medicine, Mayo Clinic Florida, USA

## Abstract

Postobstructive pulmonary edema (POPE) also known as negative pressure pulmonary edema (NPPE) is an underdiagnosed entity in clinical practice and can lead to life-threatening hypoxemia. A 64-year-old male patient's perioperative course was complicated by acute hypoxemic respiratory failure, after extubation following general anesthesia, following the excision of the right vocal cord papilloma. His chest X-ray showed features of pulmonary edema, EKG showed dynamic ST-T changes in the lateral leads, and echocardiography showed evidence of regional motion abnormalities. His coronaries were normal on the immediate angiogram. He was managed with lung protective mechanical ventilation strategy, diuretics, and fluid restriction. His respiratory status improved, and trachea was extubated after 10 hours of intensive care unit (ICU) stay. The case illustrates the various differentials of immediate postoperative flash pulmonary edema and ensuing appropriate management strategy.

## 1. Introduction

Negative pressure pulmonary edema (NPPE), a form of noncardiogenic pulmonary edema, results from marked inspiratory effort against a closed airway [1, 2]. Also known as postobstructive pulmonary edema (POPE), NPPE is first described in 1977 [3]. This condition remains unfamiliar to the medical community, making NPPE an underdiagnosed and underreported condition [2, 4, 5]. Several causes of upper airway obstruction are associated with NPPE, including airway infections or tumors in children and laryngospasm during anesthesia or following extubation in adults [6]. We report the case of a 64-year-old patient initially suspected to have acute coronary syndrome but found to have POPE on further evaluation.

## 2. Case History

A 64-year-old male underwent surgical excision of a vocal cord papilloma, under general anesthesia, and received approximately one liter of crystalloid during the procedure. His medical history was significant for compensated liver cirrhosis secondary to Hepatitis C infection, hepatocellular carcinoma, and peripheral neuropathy. His perioperative course was complicated by acute hypoxemic respiratory failure immediately following extubation. He was reintubated in the operating room, given intravenous furosemide, and transferred to the ICU for further care.

**Figure 1:**
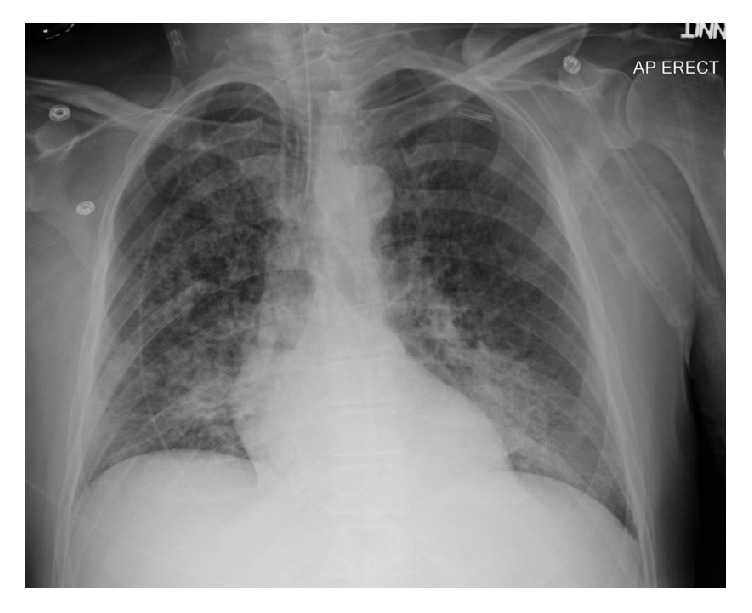
Chest X-ray showing features of pulmonary edema.

Upon ICU arrival, he was sedated with propofol and mechanically ventilated on volume control mode, with tidal volume 6ml/kg, PEEP 7cm of H_2_O, and FiO_2_ 70%. Physical exam revealed bilateral lung crepitus and normal heart sounds, with pink frothy sputum in the endotracheal tube. His chest radiograph (Figure 1) demonstrated bilateral alveolar shadows and patchy interstitial infiltrates most notable in the perihilar regions, consistent with pulmonary edema. Electrocardiogram (ECG) showed dynamic changes in both the T waves and ST segment in the lateral leads. These ECG changes, in conjunction with hypokinesis of the mid-septal and anterior walls on bedside echocardiography and hypotension, were concerning for acute coronary syndrome. Immediate coronary angiography revealed normal coronary arteries.

**Figure 2:**
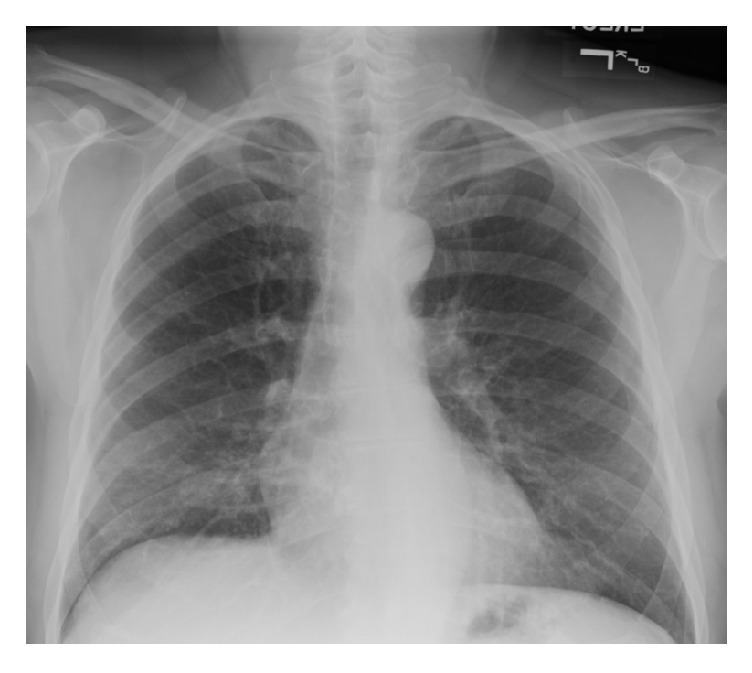
Postextubation chest x- ray showing resolution of the edema.

NPPE was suspected in this patient due to the absence of preexisting heart disease, negative cardiac workup, and recent papilloma resection. He was managed with protective mechanical ventilator support, diuretics, and fluid restriction. His respiratory status improved with resolution of the pulmonary edema (Figure 2) in the ensuing twelve hours. His subsequent hospital course was uneventful.

## 3. Discussion

This case illustrates a common clinical dilemma and frequently missed differentials of pulmonary edema in the immediate perioperative period. NPPE is estimated to occur in 1 of every 1000 postanesthesia patients, and laryngospasm is the culprit in the majority of the cases involving adults [2, 5, 6]. As exemplified in our report, the onset of NPPE is usually rapid, although in a minority of patients the initial presentation can be delayed for up to four hours [5].

The pathogenesis of NPPE is mainly attributed to the generation of marked negative intrapleural pressure [2, 5, 6]. In adults, an inspiratory effort against a closed upper airway, known as the Muller maneuver, can generate up to negative 140 mm H_2_O pressure. This negative pressure is sufficient to greatly increase the venous return to the right heart and displace the interventricular septum towards the left ventricle, decreasing the stroke volume. Simultaneously, as more blood reaches the pulmonary circulation following the increase in the venous return, the increasing hydrostatic forces in the pulmonary microvasculature favor the transudation of fluid from the vascular bed to the interstitium [1, 5, 6]. Although negative intrapleural pressure is the main component of NPPE pathogenesis, other factors also play an important role. The effort to ventilate through an obstructed airway eventually leads to hypoxia and acidosis which increases the pulmonary vascular resistance and negatively affects the alveolar capillary integrity. The marked inspiratory effort also generates a hyperadrenergic response that further increases the pulmonary vascular resistance and directly contributes to the redistribution of blood from the systemic circulation to the pulmonary circuit [5]. As illustrated in our report, a decrease in the myocardial contractility follows the hypoxic and acidotic states established in the patients with NPPE. This myocardial depression further compromises cardiac output and allows more blood to back up into the pulmonary circulation which potentiates edema formation [5].

Typical signs and symptoms of NPPE include respiratory distress, hypoxia, cyanosis, frothy pink sputum, and hemoptysis [1, 7]. This diagnosis requires a high level of suspicion from the clinician, as the presentation mimics aspiration pneumonia during anesthesia (Mendelson's syndrome) and other causes of pulmonary edema, including cardiogenic pulmonary edema and iatrogenic volume overload [2]. The radiographic findings may be useful in differentiating NPPE from cardiogenic pulmonary edema. NPPE often demonstrates marked bilateral perihilar alveolar infiltrates, while in cardiogenic pulmonary edema the infiltrates follow a more interstitial pattern and marked diversion of blood flow to the lung apices is usually seen [1, 2].

NPPE itself can promote cardiac depression in consequence to hypoxia and the subsequent acidotic state, which may make the diagnostic workup misleading [5]. Like in our case, a patient with minimal cardiovascular risk factors and no history of heart disease developed transient ECG changes and myocardial hypokinesis on echocardiogram. Further workup with a cardiac angiogram demonstrated no coronary compromise which corroborates our diagnosis of stress cardiomyopathy as part of the NPPE presentation.

The treatment of NPPE includes careful monitoring, maintenance of a patent airway, oxygen supplementation, and positive end-expiratory pressure via endotracheal intubation or noninvasive ventilation [6, 8]. Due consideration should be given to the underlying pathology responsible for the patient's edema, while considering intubation. It is advisable to follow the institutionalized difficult airway algorithm for optimal patient outcome. When mechanical ventilation is necessary, the recommendation is to perform lung protective ventilation even in patients without ARDS [8]. Despite being standard of care in cardiogenic pulmonary edema, diuretics have a secondary role in the treatment of NPPE and should be given with discretion [6]. This condition is typically self-limited with resolution of pulmonary edema and clinical improvement seen within 24–48 hours [6]. While weaning from the mechanical ventilatory support precautionary measures should be taken to ensure the resolution of edema not only from the pulmonary parenchyma but also of the upper airway. Close monitoring is needed to prevent recurrence and reintubation.

*In conclusion*, although NPPE is a life-threatening and well described postanesthesia complication, it remains an underdiagnosed event. Increased vigilance among the providers is essential to prevent the morbidity associated with this condition.

## Additional Points

*Key Messages*. NPPE should be considered in patients who develop acute respiratory distress following extubation, particularly when there is no previous history of cardiac disease or cardiovascular risk factors. Though management is primarily supportive, prompt identification of NPPE is essential to prevent catastrophic patient morbidity and mortality.
